# Chronic supplementation of a mushroom blend on oxygen kinetics, peak power, and time to exhaustion

**DOI:** 10.1186/1550-2783-12-S1-P45

**Published:** 2015-09-21

**Authors:** Katie R Hirsch, Meredith G Mock, Erica J Roelofs, Eric T Trexler, Abbie E Smith-Ryan

**Affiliations:** 1 Applied Physiology Laboratory, Department of Exercise and Sport Science, University of North Carolina at Chapel Hill, Chapel Hill, NC 27599, USA

## Background

*Cordyceps militaris *has been used in pre-workout supplement blends intended to improve aerobic performance. Mushroom blends containing *Cordyceps *may serve as an ergogenic aid by improving oxygen kinetics and delaying fatigue, but there is limited data on the effects of this ingredient during exercise. The purpose of this study was to determine the effects of 3 weeks of supplementation with a mushroom blend on oxygen kinetics, aerobic power, time to exhaustion, and lactate levels during high-intensity exercise.

## Methods

In a double-blind placebo controlled design, recreationally active subjects (n = 10; mean ± SD; age = 21.4 ± 2.4 yrs; height = 175.8 ± 78 cm; weight = 75.0 ± 10.6 kg) were randomly assigned to a mushroom (MR; n = 6) or placebo (PL; n = 5) treatment group. All subjects completed a maximal graded exercise test, 6 min sub-maximal cycle test, and 3 min all-out cycle test, each separated by at least 24 hrs. Maximal oxygen consumption (VO_2 _max), time to exhaustion (TTE), and ventilatory threshold (VT) were determined via respiratory gas analysis during the maximal graded exercise test performed on a cycle ergometer. Lactate and oxygen saturation (SPO_2_) were measured at 0, 2, 3 and 6 min during the 6 min sub-max cycle test at a workload of 60% between VT and VO_2 _max. Peak power output (PP) was recorded during the 3 min all-out cycle test with a resistance of 4.5% of body weight. Subjects were given capsules containing either 1.3 grams of mushroom blend or 1.3 grams of maltodextrin (PL) to be taken 3 times per day (4 grams daily) for 3 weeks. The same 3 exercise tests were repeated after 3 weeks.

## Results

There was a significant increase in VO_2 _max (44.0 ± 10.5 to 48.8 ± 11.1 ml/kg/min^-1^; p = 0.042) for MR. There was also an increase in TTE (+69.8 sec; 851.7 ± 170.0 to 921.5 ± 146.2 sec) for MR as determined by 95% confidence intervals. No changes in VO_2 _max or TTE were observed for PL (p > 0.05). Though not statistically significant (p > 0.05), there was a greater increase in VT for MR (+0.9 L/min; 1.7 ± 0.3 to 2.4 ± 1.0 L/min) compared to PL (+0.2 L/min; 2.3 ± 0.9 to 2.5 ± 0.7 L/min). Lactate increased significantly over the 6 min test in both groups with no significant difference between groups (p = 0.369). There was also a non-significant increase (p > 0.05) in PP in MR (51 ± 113 W) and a decrease in PP in PL (- 48 ± 50 W).

## Conclusion

Chronic, 3 week supplementation of a mushroom blend at 4 grams per day may improve VO_2 _max_, _increase TTE and augment PP during high-intensity aerobic exercise. A blend containing *Cordyceps militaris *may be an effective method for enhancing aerobic performance and delaying fatigue by improving oxygen kinetics. This could have positive implications for maintaining and improving training volume, especially in endurance athletes.

